# Polyploid giant cancer cells and tumor budding: translation from basic research to clinical application

**DOI:** 10.3389/fonc.2025.1611920

**Published:** 2025-07-16

**Authors:** Peng Huang, Rong Wu, Zhimou Yang, Yuwei Li, Fei Fei, Yongjun Yu

**Affiliations:** ^1^ Nankai University School of Medicine, Nankai University, Tianjin, China; ^2^ Department of Scientific and Technology, Nanjing First Hospital, Nanjing Medical University, Nanjing, Jiangsu, China; ^3^ Key Laboratory of Bioactive Materials, Ministry of Education, State Key Laboratory of Medicinal Chemical Biology, College of Life Sciences, Nankai University, Tianjin, China; ^4^ Department of Colorectal Surgery, Tianjin Union Medical Center, The First Affiliated Hospital of Nankai University, Tianjin, China; ^5^ Department of Oncology, Nanjing First Hospital, Nanjing Medical University, Nanjing, Jiangsu, China

**Keywords:** tumor budding, metastasis, drug resistance, therapeutic strategies, polyploid giant cancer cells (PGCCs)

## Abstract

Polyploid giant cancer cells (PGCCs) represent a distinct subpopulation of tumor cells characterized by enlarged or multiple nuclei and aneuploidy. PGCCs are products of genomic instability, possessing cancer stem cell properties and exhibiting significant resistance to radiotherapy and chemotherapy. They can generate highly invasive daughter cells through asymmetric division, exhibiting epithelial-mesenchymal transition characteristics, and facilitating tumor recurrence and metastasis. *In vivo*, PGCCs with daughter cells in tumor tissue can migrate and infiltrate into the forefront stroma to form tumor budding, which are closely related to solid tumor recurrence, metastasis, and drug resistance. Studies have shown that inhibiting sphingolipid enzyme acid ceramidase or regulating autophagy can reduce the production of PGCCs with daughter cells. Under appropriate induction conditions, PGCCs with daughter cells can be induced to differentiate into benign tissues such as adipocytes, chondrocytes, and osteocytes, inhibiting their malignant proliferation and invasive destruction. This study reviewed the recent research developments regarding PGCCs, mainly explored the endogenous mechanisms of PGCCs formation and their malignant phenotype, as well as the process of tumor budding formation *in vivo* and potential therapeutic strategies targeting PGCCs. The main novelty of this study lies in exploring the translation of PGCCs basic research into the clinical pathological prognostic role of tumor budding, which can reveal the potential mechanism of PGCCs/tumor budding formation at the molecular level, providing theoretical basis for prognosis assessment, monitoring of recurrence and metastasis risks, as well as improving drug resistance and targeted therapy in cancer patients.

## 1 Introduction

Polyploid giant cancer cells (PGCCs) have emerged as a critical area of investigation in cancer research. These cells exhibit distinctive characteristics including abnormally enlarged cell and nuclear size, abundant cytoplasm, significant nuclear pleomorphism, high nuclear-cytoplasmic ratio, deep nuclear staining, prominent nucleoli, and either mono- or multinucleated structures (1, 2). PGCCs have been documented in numerous solid tumors, including breast cancer, ovarian cancer, colorectal cancer, lung cancer, pancreatic cancer, bladder cancer, renal cancer, thyroid cancer, and prostate cancer (3–5). These cells have been identified in the urine of prostate cancer patients (6). Research has also revealed the presence of polyploid giant cells in leukemia patients (7, 8). Based on the important role of PGCCs in the occurrence and development of malignant tumors, this study mainly reviewed the molecular mechanisms of the formation and high invasion and metastasis characteristics of PGCCs, as well as potential therapeutic strategies targeting PGCCs.

**Figure 1:**
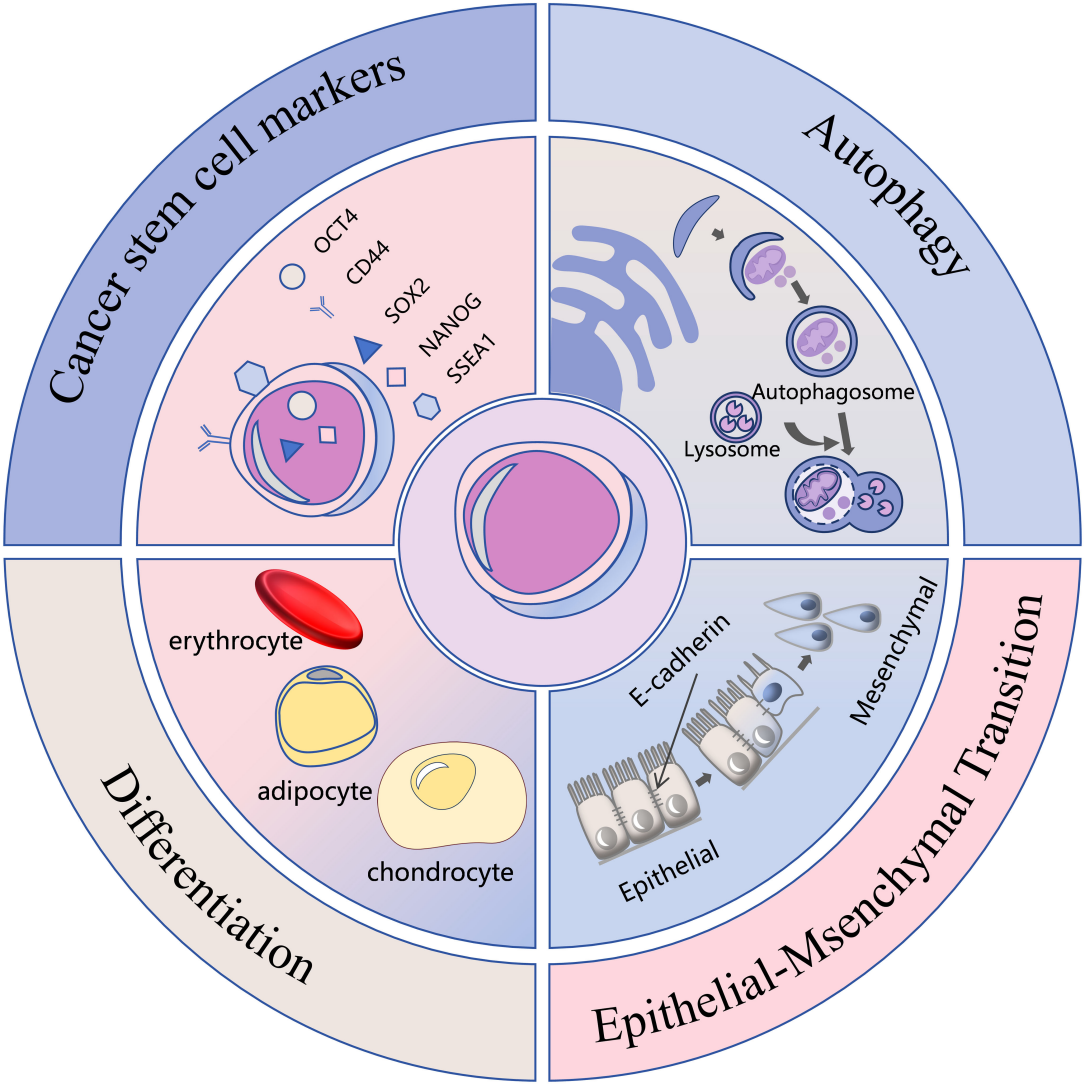
Malignant phenotype and endogenous mechanisms of PGCCs.

PGCCs demonstrate strong associations with multiple malignant characteristics of cancer, including drug resistance, recurrence, metastatic potential, and tumor microenvironment remodeling (1, 2). These cells exhibit cancer stem cell properties, demonstrating robust proliferative capacity and the ability to differentiate into benign tissues such as erythrocyte, adipocyte, chondrocyte, and bone (9, 10). They express cancer stem cell markers including CD44, OCT4, SOX2, NANOG and SSEA1 (11, 12). Additionally, PGCCs can undergo epithelial-mesenchymal transition (EMT) (13), avoid drug-induced apoptosis through autophagy activation (14), and display Warburg effect metabolism (15), establishing them as significant targets in cancer therapy research. Their high heterogeneity and complex biological behavior, however, present ongoing challenges for comprehensive understanding. The malignant phenotype and endogenous mechanisms of PGCCs are shown in **Figure 1**.

Recent advances in single-cell omics, high-resolution microscopy imaging, and functional genomics have facilitated enhanced understanding of PGCC origin, formation mechanisms, and functions within the tumor ecosystem. This study mainly reviewed the recent research developments regarding PGCCs, discussed the internal mechanism of their formation and high invasion and metastasis characteristics, as well as the process of tumor budding formation *in vivo* and potential therapeutic strategies targeting PGCCs, while considering current challenges and future research directions.

## 2 Formation mechanisms of PGCCs

**Figure 2:**
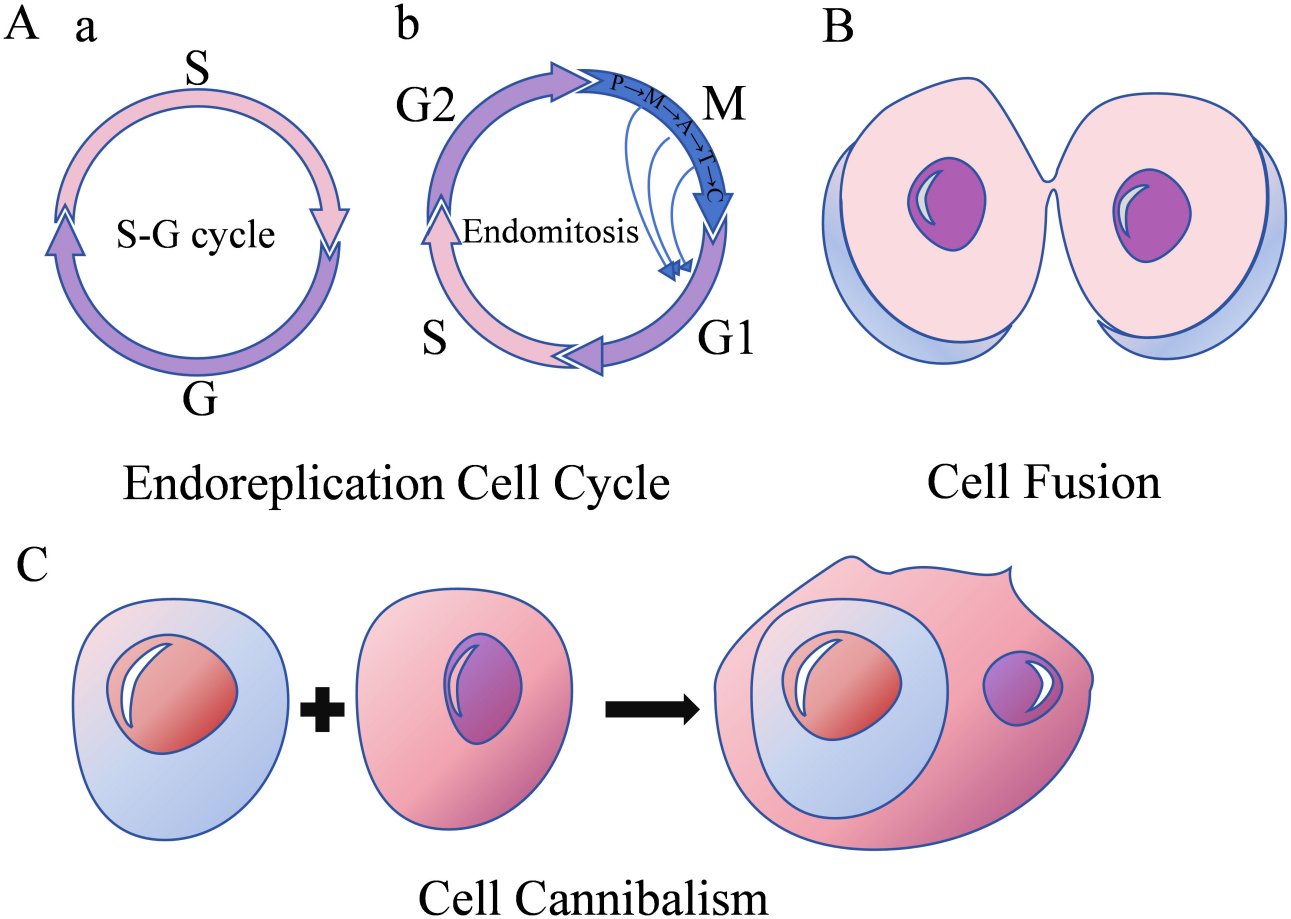
Formation mechanisms of PGCCs. **(A)** Endoreplication cell cycle with two subfigures; a shows an S-G cycle, and b shows endomitosis including G1-S-G2-M phases. **(B)** Cell fusion depicted as two cells merging. **(C)** Cell cannibalism.

Numerous researches have established that various stimuli, including hypoxia (16), chemotherapy (17), radiotherapy (18), tumor microenvironment alterations (13), gene mutations (such as SF3B1, p53) (7, 19), pesticides (20), and viral infections (21–26), can induce PGCC formation, leading to their entry into a distinctive life cycle known as the polyploid giant cell cycle (27). The formation mechanism of PGCCs is shown in **Figure 2**.

### 2.1 Endoreplication cell cycle

The conventional mitotic cell cycle comprises G1-S-G2-M phases. Cell cycle progression requires activation of both synthesis-cyclin dependent kinase (S-CDK) during the synthesis phase and mitosis-cyclin dependent kinase (M-CDK, comprising CDK1, cyclin A, and cyclin B) during mitosis, with their combined CDK levels reaching a specific threshold (16). Endoreplication refers to genome replication occurring without cell division, maintaining nuclear membrane integrity while doubling or further increasing genomic content. Two distinct forms of endoreplication exist, differentiated by whether cells enter mitosis. The first form is the endocycle, characterized by alternating S and G phases (S-G cycle) (2), without chromosome and cell separation. During this process, M-CDK expression levels decrease, extending the G2 phase and preventing cell cycle progression into the M phase. Simultaneously, periodic S-CDK inactivation enables alternating G and S phase progression. Tumor cells entering the endocycle form polyploid tumor giant cells. The second form of endoreplication is endomitosis, wherein cells pass the G2/M checkpoint and undergo abortive mitosis without complete sister chromatid separation or cell division (16), resulting in either a single giant nucleus, a lobulated nucleus, or a polyploid containing multiple small nuclei.

### 2.2 Cell fusion

Cell fusion occurs through dynamic interactions between the cell membrane and cytoskeleton. Fusion can occur between cells of the same type (homotypic fusion) or different types (heterotypic fusion) (1). This process induces chromosomal instability, DNA damage, and uneven distribution of genetic material, thereby promoting PGCC formation and contributing to tumor development, progression, drug resistance, and metastasis.

### 2.3 Cell cannibalism

Cell cannibalism refers to the process where one cell engulfs another, leading to the death of the internalized cell. The internalized cell functions as a physical barrier within the host cell cytoplasm, preventing cytoplasmic division and resulting in polyploidy (17).

## 3 Invasion and metastasis of PGCCs with their daughter cells

The enhanced invasive and metastatic capabilities of PGCCs with their daughter cells primarily depend on traction and polarity. Actin generates traction on the surrounding tumor microenvironment, propelling the cell in the direction of movement. PGCCs exert maximum traction forces 2 to 5 times higher than non-PGCCs. Beyond traction, movement polarity or directionality is crucial. Units with persistent paths and directionality traverse longer distances than those moving randomly. Intermediate filament proteins serve as key drivers of cell polarization, playing essential roles in anterior-posterior cell polarization (28).

Vimentin, an atypical nuclear scaffold protein, demonstrates significant functions in PGCCs. Fan L et al. (29) reported that vimentin’s nuclear localization enhances nuclear stability and promotes PGCC invasion by activating EMT-related signals. The small ubiquitin-like modification (SUMOylation) of vimentin facilitates cell proliferation and migration, increasing cancer cell proliferation and invasiveness. Furthermore, vimentin functions as a transcription factor promoting daughter cell migration through the vimentin-ARHGAP10-CDC42-cathepsin B and D signaling pathway (29).

PLK4 and Cdc42 signaling represent crucial regulatory factors for PGCC generation and invasion. PLK4, a key centrosome regulatory protein, enhances microtubule organization when upregulated, promoting PGCC migration and division (30). Additionally, Cdc42 regulates cytoskeleton reorganization, enabling PGCCs to penetrate the basement membrane and invade adjacent tissues (11). Increased Cdc42 and PAK1 expression reduces STMN1 expression while increasing phosphorylated STMN1, a protein located in PGCC nuclei and daughter cells, regulating cytoskeleton remodeling. Low PTPN14 expression, a downstream regulatory protein of STMN1, confers invasive and metastatic capabilities to PGCC with daughter cells.

**Figure 3:**
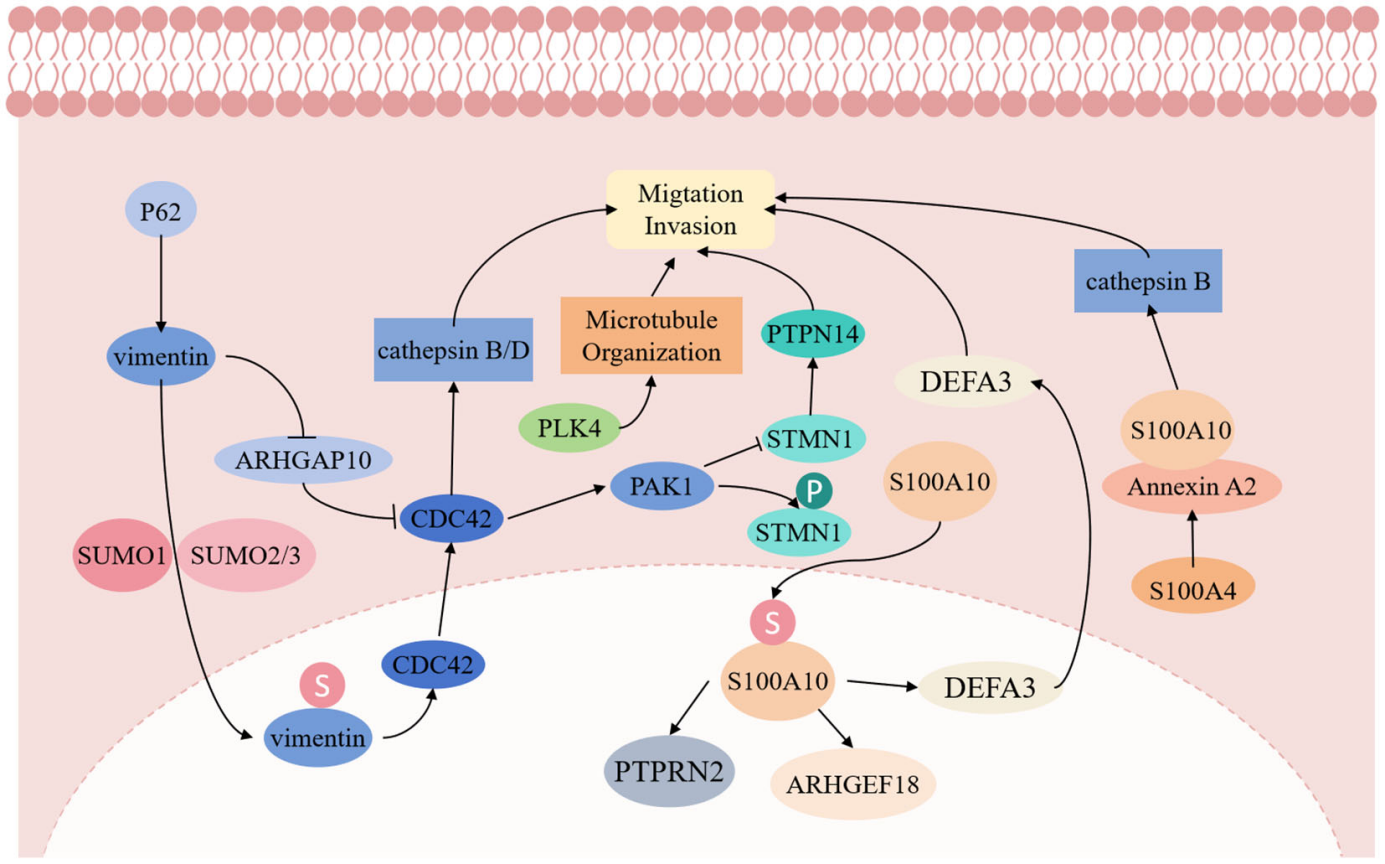
The molecular mechanism of invasion and metastasis of PGCCs with their daughter cells.

The S100A4 gene plays a vital role in PGCC invasion and metastasis. Fei F et al. (31) demonstrated that PGCC with the daughter cell migration, invasion, and proliferation capabilities were significantly inhibited after S100A4 knockout. S100A4 regulates the Annexin A2/S100A10 complex structure and function, affecting downstream cathepsin B, leading to PGCC and daughter cell invasion and metastasis. Additionally, S100A10 can undergo nuclear transport via SUMOylation modification, regulating ARHGEF18, PTPRN2, and DEFA3 expression, thereby promoting PGCC and daughter cell proliferation, migration, and invasion (32). The molecular mechanism of invasion and metastasis of PGCCs with their daughter cells is shown in **Figure 3**.

## 4 PGCCs with daughter cells infiltrate into the stroma of the tumor tissue front to form tumor budding

Tumor budding represents a significant pathological parameter, characterized by single tumor cells or clusters of up to four tumor cells located at the invasive front of the tumor (33). The 2016 International Tumor Budding Consensus Conference established a three-grade classification system: Bd1 (0-4 buds), Bd2 (5-9 buds), and Bd3 (≥10 buds) (34). Tumor budding serves as an independent predictor of lymph node metastasis in pT1 colorectal cancer and survival in stage II colorectal cancer (35). Studies demonstrate that intermediate and high-grade tumor budding correlate with significantly lower 5-year disease-specific survival rates (52%-80%) compared to low-grade tumor budding (89%-98%) (33). Additionally, tumor budding assessment in colorectal cancer biopsies aids in treatment planning for rectal cancer patients following neoadjuvant therapy (36, 37). Beyond its clinical predictive role in colorectal cancer, tumor budding functions as a prognostic biomarker across various cancers, including head and neck squamous cell carcinoma (38, 39), gastric cancer (40), lung cancer (41), giant cell tumor of bone (42), and breast cancer (43). Research consistently demonstrates that high-grade tumor budding correlates with increased lymph node metastasis and decreased overall survival. However, certain studies indicate that the tumor budding score shows significant correlation with tumor stage (*P*<0.001), lymph node metastasis (*P*<0.05), and distant metastasis (*P*<0.05) in lung squamous cell carcinoma, but not with overall survival rate, tumor size, or pleural invasion (44).

**Figure 4:**
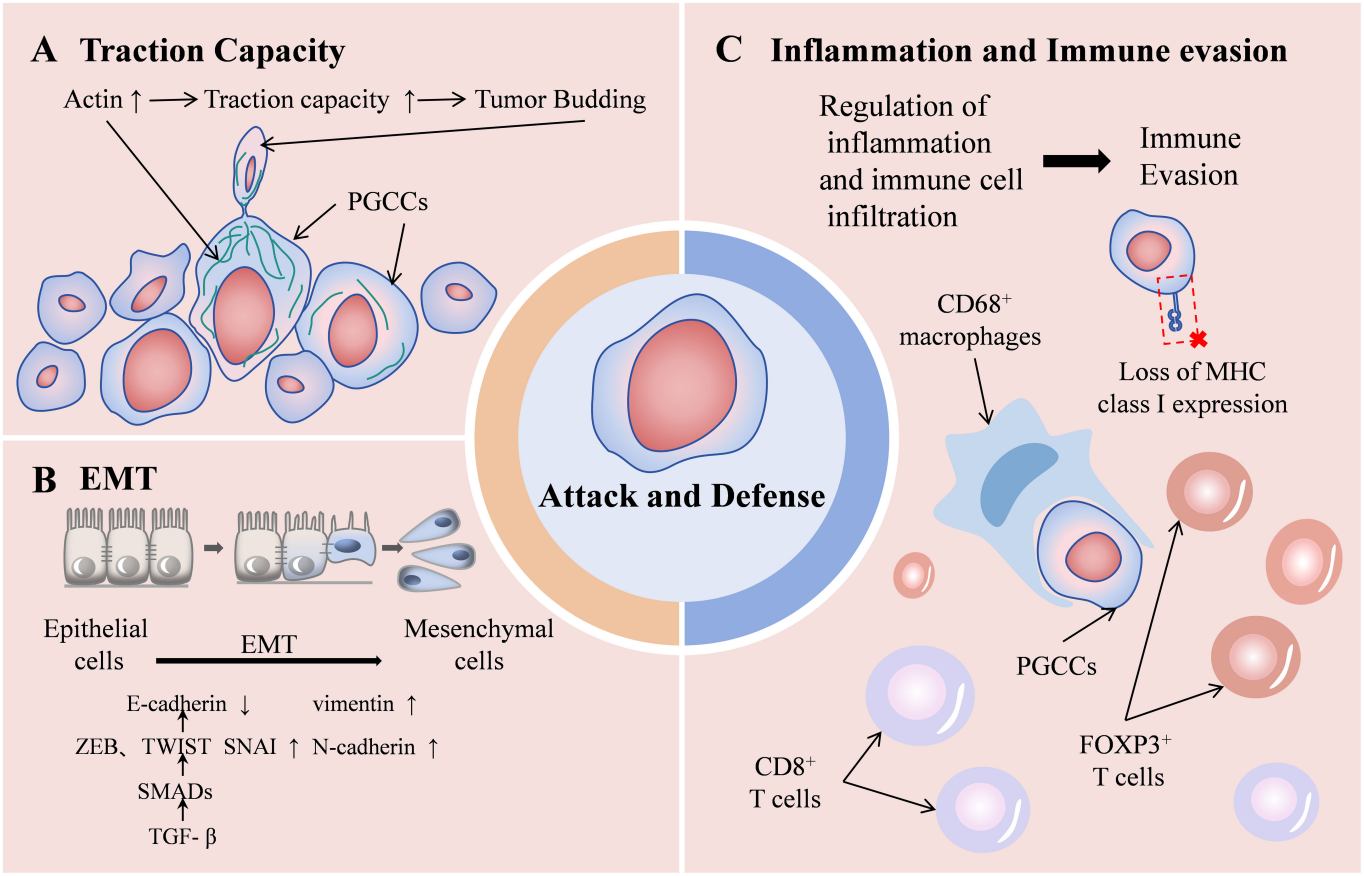
Schematic diagram of the “attack-and-defense” dynamic of PGCCs/tumor budding. Enhanced traction capacity contributing to tumor budding **(A)** and EMT process **(B)** represent the attack state, while the role of promoting inflammation and immune evasion **(C)** represents the defense state.

Research has explored combining tumor budding with other indicators as independent prognostic factors. The Tertiary Lymphoid Structures/Tumor Budding index, serves as an independent prognostic factor for triple-negative breast cancer (45). Combining tumor budding and tumor-stroma ratio enhances prognostic stratification in colon cancer patients (34). Furthermore, the integration of tumor budding and immune score provides superior prognostic prediction for pTNM I-III stage colon cancer patients compared to individual factors (46). Tumor budding exhibits relative uniformity and operates independently of tumor grading or other morphological features (47). It represents the initial phase of tumor metastasis, wherein tumor cells undergo EMT process, migrate into the extracellular matrix, infiltrate lymphatic and vascular tissues, and establish metastatic colonies in lymph nodes or distant locations (35). This process involves loss of cell polarity and deterioration of cell connections, including adhesion and gap junctions. Matrix metalloproteinases degrade the extracellular matrix, accompanied by downregulation of cell surface proteins such as E-cadherin and upregulation of N-cadherin. The TGF-β signaling pathway participates through SMAD phosphorylation, inducing ZEB, TWIST, and SNAIL family members, thereby suppressing E-cadherin transcription (48). These alterations collectively facilitate tumor budding. EMT functions as an immune evasion mechanism, where tumor cells lose major histocompatibility complex expression, becoming undetectable to effector immune cells. The interaction between tumor budding and the immune system manifests as an attack-defense model: tumor budding indicates an invasive phenotype, while CD8+ T cells mediate anticancer responses (33) (This “attack-and-defense” dynamic as shown in **Figure 4**). However, at present, tumor budding is only a pathological change, which is limited by the lack of *in vitro* research models, and its formation mechanism and the internal regulation mechanism of malignant phenotype are still unclear.

The appearance of PGCCs in tumor tissues occurs through two primary mechanisms. First, PGCCs may pre-exist in tumor tissue, arising from intrinsic epigenetic factors and extrinsic exposure factors (including smoking, high temperature, ultraviolet light) that modify the tumor microenvironment (hypoxia, immune modulation), triggering tumor cell dedifferentiation and PGCC formation (3). Second, during chemotherapy, radiotherapy, or targeted therapy, external factors stimulate tumor cells to undergo endoreplication cell cycle, cell fusion, or cell cannibalism, leading to multiple genome replications without cell division, resulting in PGCC formation and subsequent drug resistance. These adaptations enable cancer cells to persist under adverse conditions while evading immune surveillance and developing treatment resistance (17). PGCCs maintain their self-renewal through endoreplication and subsequently divide via nuclear budding or fission, generating highly invasive progeny cells through EMT, which contributes to drug resistance and tumor recurrence. In the first pathway, PGCCs situated outside the tumor focus undergo EMT, gaining enhanced migration and invasion capabilities, marked by the reduction of epithelial markers including E-cadherin and the acquisition of mesenchymal markers such as vimentin and N-cadherin, or the expression of transcription factors like SNAI1 and SNAI2 (13). These alterations enable the PGCC daughter cells to penetrate the basement membrane and establish distant metastases.

**Figure 5:**
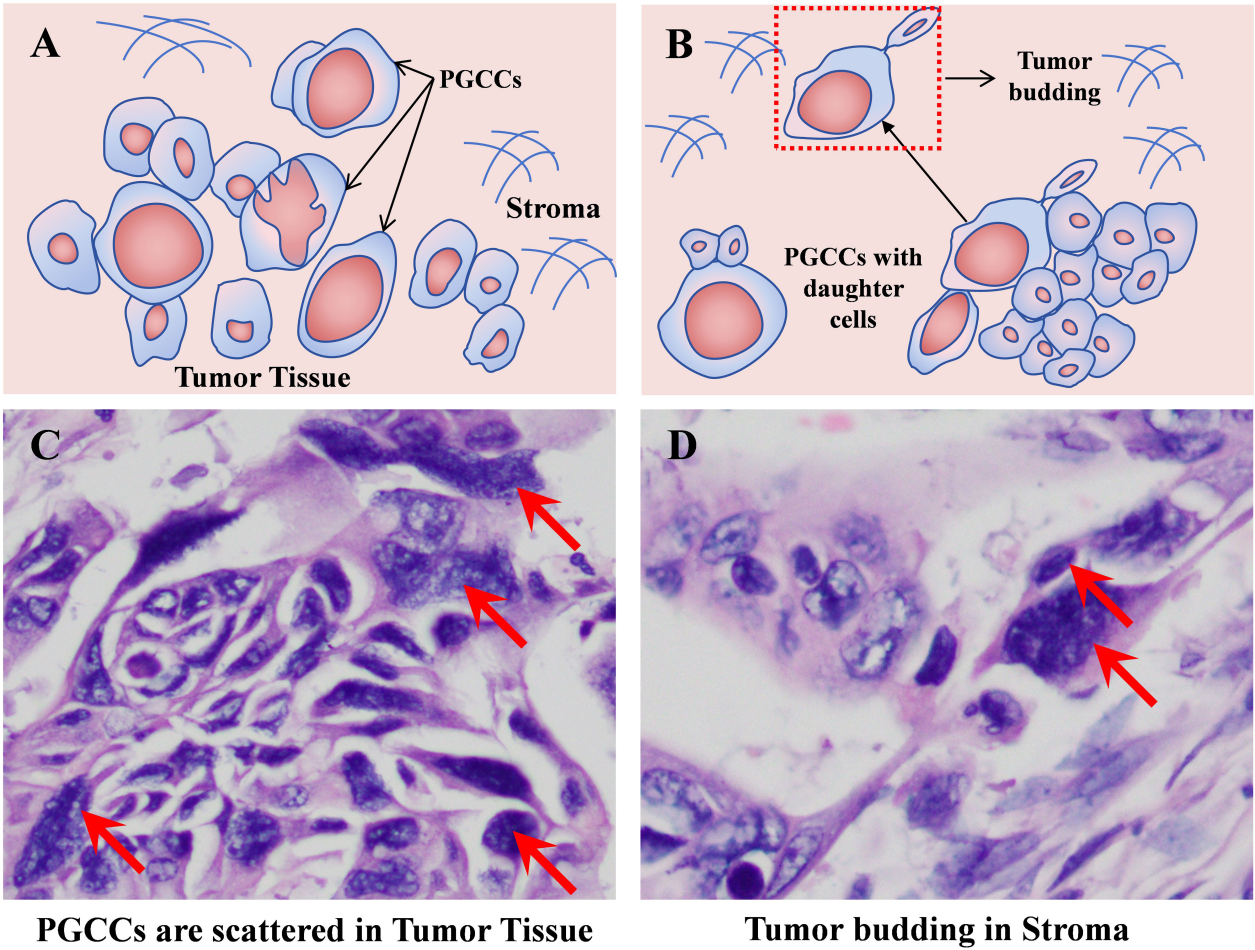
PGCCs with daughter cells infiltrate into the stroma of the tumor tissue front to form tumor budding. **(A)** Pattern diagram of PGCCs scattered in tumor tissue. **(B)** Pattern diagram of tumor budding formed by PGCCs with daughter cells. **(C)** Solid tumor tissue H&E staining (red arrow indicating PGCCs, 400×). **(D)** H&E staining of tumor budding (red arrow indicating tumor budding, 400×). PGCCs, polyploid giant cancer cells.

Based on the aforementioned studies, it is not difficult to find the process where PGCCs with their daughter cells acquire EMT characteristics, migrate, and infiltrate into the frontier stroma, forming tumor budding (as shown in **Figure 5**). Therefore, as a superior *in vitro* research model, further exploration of the endogenous mechanisms underlying the formation and high invasive characteristics of PGCCs with their daughter cells holds promise for providing theoretical evidence at the molecular level for prognosis assessment, recurrence and metastasis risk monitoring, and precision treatment in early-stage cancer patients.

## 5 Potential therapeutic strategies based on PGCCs with their daughter cells

Sphingolipid enzyme acid ceramidase (ASAH1) can interfere with the generation of PGCCs with their daughter cells. ASAH1 is situated in lysosomes, where it hydrolyzes ceramide to generate sphingosine. Sphingosine, serving as a substrate for sphingosine kinase 1 or 2, gives rise to sphingosine-1-phosphate (S1P) by the addition of a phosphate group. Ceramide and S1P are functionally antithetical. The former is generally considered to be pro-apoptotic, while the latter is involved in survival, mitosis, and angiogenesis (49). Hence, it is commonly held that the conversion of ceramide into sphingosine by ASAH1 will shift the intracellular balance from apoptosis to a pro-survival state. White-Gilbertson et al. found that UC2288 can impede the expression of p21, thereby reducing the expression of acid ceramidase and inhibiting the generation of PGCCs and their daughter cells (50). In another study by White-Gilbertson et al., it was revealed that tamoxifen can disrupt the function of ASAH1 through off-target effects, thus precluding the generation of PGCCs with daughter cells (51). In addition, LCL521 can also inhibit ASAH1, resulting in the accumulation of ceramide on the surface of PGCCs and preventing the formation of their daughter cells. This study also uncovered that the accumulation of ceramide appears to supplant cholesterol on the cell surface. The competition between cholesterol and ceramide on the plasma membrane may affect membrane fluidity and the ability of PGCCs to generate progeny through amitosis (52).

Autophagy constitutes a conserved self-digestion process that eliminates damaged and superfluous organelles, misfolded or aggregated proteins, and intracellular pathogens, playing a crucial role in maintaining cellular homeostasis under stress conditions (14). You B et al. (53) established that autophagy-dependent PGCC formation results from AMPK-mTOR pathway activation, with RIPK1 functioning as a scaffold protein to promote PGCC survival through this pathway. This investigation pioneered the identification of RIPK1’s protective role in dormant cell survival, presenting a novel opportunity for targeted PGCC treatment. Further research indicated that autophagy-regulating drugs significantly impair ovarian cancer PGCC colony formation capabilities. Thus, autophagy emerges as a promising treatment target to prevent PGCC tumor regeneration (54).

Beyond these treatment strategies, intervention can occur through modulation of tumor cell metabolism or promotion of PGCC cell death. Research has shown that zoledronic acid application reduces lipid droplet and cholesterol content, mitochondrial density, and ROS in PGCCs, effectively eliminating PGCCs (55). The microtubule-targeting agent ST-401 triggers a transient integrated stress response, decreases energy metabolism, promotes mitochondrial fission, and subsequently induces interphase cell death while preventing PGCC formation (56). Ferroptosis inducers demonstrate effectiveness in eliminating breast cancer PGCCs with low expression of ferroptosis regulators (57).

Research has demonstrated that combining mifepristone and olaparib synergistically inhibits PGCC endoreplication, yielding enhanced antitumor efficacy compared to individual drug administration (58). While PRL3 induces PGCC formation, PRL3-zumab prevents PGCC formation and tumor recurrence through PRL3 targeting and inhibition (59). IL-1β inhibition reduces senescence-related protein p-histone H2A.X expression and enhances the pro-apoptotic effect of docetaxel (60). IL-6 facilitates PGCC formation, embryonic stemness acquisition, and fibroblast transformation into tumor-associated fibroblasts, contributing to chemotherapy resistance (61). In addition, studies have shown that PGCCs represent a critical factor in solid tumor immunotherapy inefficacy. IL-33 induces polyploidy and immune suppression. Blocking IL-33 can induce antitumor immunity in IL-33-positive mice, increasing tumor-specific CD8+ T cell numbers (27). Furthermore, PGCCs modulate the tumor microenvironment (TME) to promote breast cancer metastasis and chemoresistance. Targeting PGCCs can improve the tumor immune microenvironment, promoting T cell survival within the PGCC-induced TME and preventing T cell functional inactivation, thereby enhancing immunotherapy efficacy (62).

**Figure 6:**
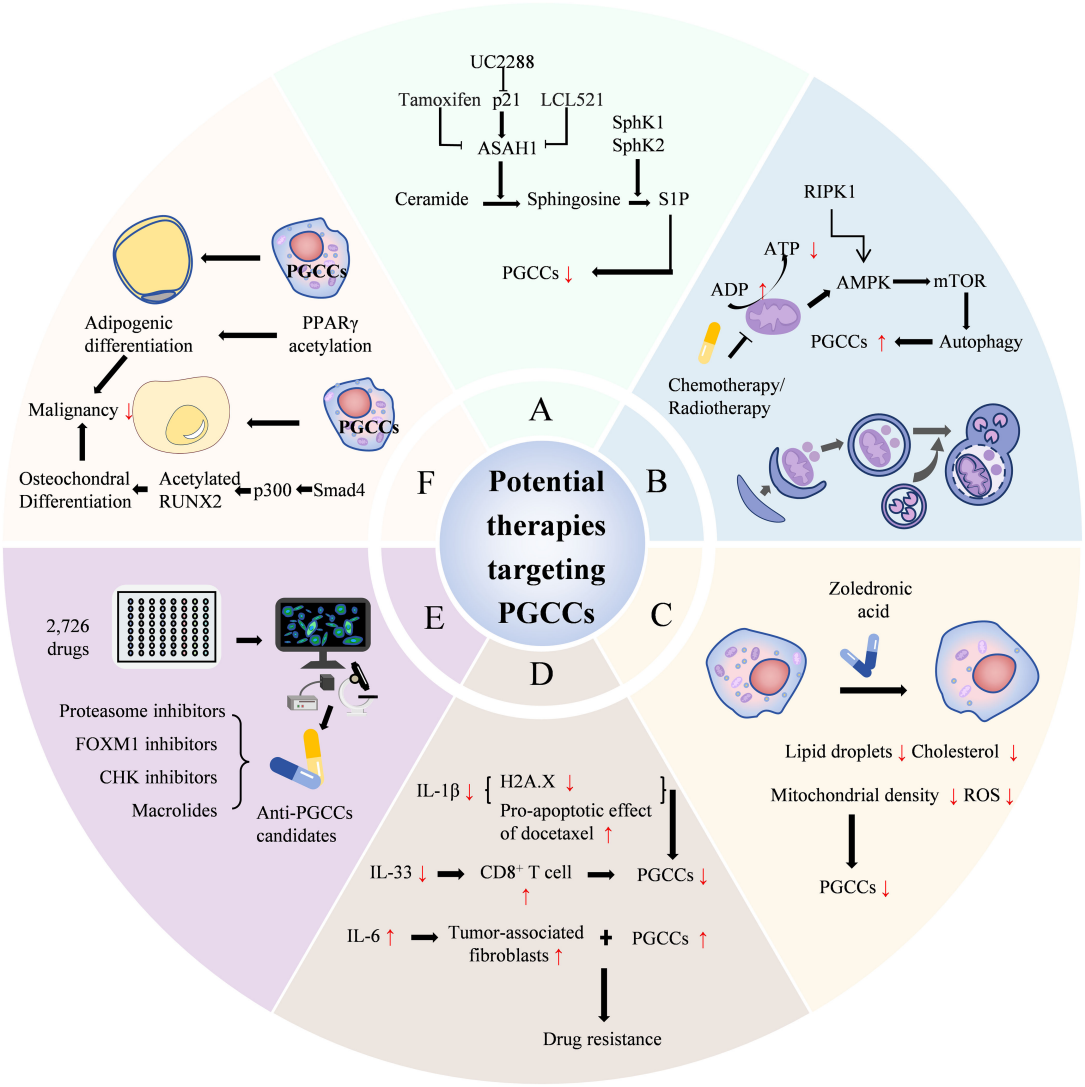
Potential therapeutic strategies based on the formation and highly invasive mechanisms of PGCCs with their daughter cells. **(A)** lists treatments like UC2288, Tamoxifen, and others targeting ceramide pathways. **(B)** shows chemotherapy and radiotherapy increasing autophagy in PGCCs. **(C)** illustrates Zoledronic acid reducing lipid droplets and cholesterol. **(D)** focuses on drug resistance involving IL-1β, docetaxel, IL-33, and CD8+ T cells. **(E)** indicates screening of drugs and inhibitors like proteasome and FOXM1 inhibitors. **(F)** describes differentiation therapies, including adipogenic differentiation and acetylated RUNX2 reducing malignancy. PGCCs, Polyploid giant cancer cells; ASAH1, Sphingolipid enzyme acid ceramidase; S1P, Sphingosine-1-phosphate.

Recently, researchers developed a high-throughput single-cell morphological analysis workflow and trained a machine learning model to identify compounds selectively inhibiting non-PGCCs, PGCCs, or both. The model evaluated 2,726 FDA Phase I-approved drugs, identifying promising anti-PGCC candidates, including proteasome inhibitors, FOXM1 inhibitors, CHK inhibitors, and macrolides. It also predicted effective compounds from over 6,000 drugs. Five top-ranked predictions were experimentally validated as effective PGCC inhibitors using cell lines and patient-derived models. These results demonstrate the potential of combining high-throughput empirical screening with machine learning-based virtual screening for accelerated therapy discovery (63). Beyond PGCC formation inhibition, inducing their differentiation into benign tissues to suppress malignant biological behavior represents a significant future cancer treatment direction. In anaerobic conditions, PGCCs undergo mesenchymal transformation and acquire enhanced cellular plasticity, generating functional cells from different lineages, including adipocytes, chondrocytes, and osteocytes. Research confirms that P300 promotes RUNX2 acetylation (64), facilitating osteochondral differentiation. Post-osteochondral differentiation, PGCCs and their daughter cells exhibit significantly reduced stemness, migration, invasion, and proliferation, creating a therapeutic window for further treatment. PPARγ acetylation promotes adipogenic differentiation of PGCCs and their daughter cells, reducing invasion, metastasis, and proliferation (65). Based on the molecular mechanisms of PGCCs with their daughter cells formation and high invasion, potential therapeutic strategies are shown in **Figure 6**.

## 6 Conclusion

PGCCs constitute a complex yet essential cell population in tumor biology, demonstrating distinctive mechanisms of tumor adaptation and malignant evolution. The main novelty of this study lies in exploring the translation of PGCCs basic research into the clinical pathological prognostic role of tumor budding, and revealing the potential mechanism of PGCCs/tumor budding formation at the molecular level, providing theoretical basis for prognosis assessment, monitoring of recurrence and metastasis risks, as well as improving drug resistance and targeted therapy in cancer patients. Nevertheless, the substantial heterogeneity and complexity inherent to PGCCs present formidable challenges for both research endeavors and clinical implementation. In the future, we need to comprehensively understand the molecular signaling and potential therapeutic strategies of PGCCs through interdisciplinary collaboration and pioneering research approaches, and promote the clinical application of PGCC-targeted interventions in malignant tumors.
